# Blends of Carbohydrate Polymers for the Co-Microencapsulation of *Bacillus clausii* and Quercetin as Active Ingredients of a Functional Food

**DOI:** 10.3390/polym14020236

**Published:** 2022-01-07

**Authors:** María Z. Saavedra-Leos, Manuel Román-Aguirre, Alberto Toxqui-Terán, Vicente Espinosa-Solís, Avelina Franco-Vega, César Leyva-Porras

**Affiliations:** 1 Coordinación Académica Región Altiplano (COARA), Universidad Autónoma de San Luis Potosí, Matehuala, San Luis Potosi 78700, Mexico; zenaida.saavedra@uaslp.mx; 2 Centro de Investigación en Materiales Avanzados S.C., CIMAV, Miguel de Cervantes No. 120, Complejo Industrial Chihuahua, Chihuahua 31136, Mexico; manuel.roman@cimav.edu.mx; 3 Centro de Investigación en Materiales Avanzados S.C., Unidad Monterrey, Alianza Norte No. 202, Parque de Investigación e Innovación Tecnológica, Apodaca 66600, Mexico; alberto.toxqui@cimav.edu.mx; 4 Coordinación Académica Región Huasteca Sur, Universidad Autónoma de San Luis Potosí, Carretera Tamazunchale-San Martin Km. 5. Tamazunchale, San Luis Potosi 79960, Mexico; vicente.espinosa@uaslp.mx; 5 Facultad de Ciencias Químicas, Universidad Autónoma de San Luis Potosí, San Luis Potosi 78210, Mexico; avelina.franco@uaslp.mx

**Keywords:** carbohydrate polymers blends, functional food, antioxidant activity, co-microencapsulation, spray drying, bacteria viability (*Bacillus clausii*), probiotics

## Abstract

A functional food based on blends of carbohydrate polymers and active ingredients was prepared by spray drying. Inulin (IN) and maltodextrin (MX) were used as carrying agents to co-microencapsulate quercetin as an antioxidant and *Bacillus clausii* (Bc) as a probiotic. Through a reduced design of experiments, eleven runs were conducted and characterized by scanning electron microscopy (SEM), X-ray diffraction (XRD), and modulated differential scanning calorimetry (MDSC). The physical characterizations showed fine and non-aggregated powders, composed of pseudo-spherical particles with micrometric sizes. The observation of rod-like particles suggested that microorganisms were microencapsulated in these particles. The microstructure of the powders was amorphous, observing diffraction peaks attributed to the crystallization of the antioxidant. The glass transition temperature (Tg) of the blends was above the room temperature, which may promote a higher stability during storage. The antioxidant activity (AA) values increased for the IN-MX blends, while the viability of the microorganisms increased with the addition of MX. By a surface response plot (SRP) the yield showed a major dependency with the drying temperature and then with the concentration of IN. The work contributes to the use of carbohydrate polymers blends, and to the co-microencapsulation of active ingredients.

## 1. Introduction

Functional foods are defined as those products consumed as part of a normal diet with additionall health benefits beyond the traditional nutrients [1], reducing the risk of suffering from chronic diseases [2]. The incorporation of active compounds such as probiotics and antioxidants is currently one of the different strategies used to generate these type of foods, [3]. Functional foods contain bioactive compounds in low concentrations, such as antioxidants, glutamine, fatty acids, and even live microorganisms as probiotics. Probiotics are defined as live strains of strictly selected microorganisms, that when consumed in adequate amounts, confer a beneficial effect to the health [4]. The effects of probiotics can vary depending on the dose, the strain used and the components which the final product was formulated. These products can contain one or more strains, such as *Lactobacillus*, *Bifidobacterium*, *Lactococcus*, *Streptococcus*, *Enterococcus*, Gram-positive strains of the genus *Bacillus*, and yeasts of the genus *Saccharomyces* [5]. Another type of bioactive compounds are antioxidants that have demonstrated the ability to protect, delay cellular aging, and strengthen the immune system [6,7]. Antioxidants are compounds that inhibit unstable free radicals that lead to a chain reaction of cellular damage, causing cellular aging and chronic degenerative diseases. However, the use of these antioxidants in the food industry is limited, since they exhibit high sensitivity to environmental conditions such as light, oxygen, humidity and exposure to heat, causing a decrease or loss of their functional properties. In order to overcome these drawbacks, it is necessary implementing technologies to minimize the loss of functional and nutritional properties of these compounds. Microencapsulation is one of the strategies employed to preserve and extend the shelf life of foods containing bioactive ingredients [8]. Microencapsulation consists of mechanical and physicochemical processes that trap the functional substance or bioactive compound within the walls of another material acting as a protective barrier. Among the encapsulation techniques, spray drying is one of the simplest, cheapest, and fastest methodologies used by both the pharmaceutical and food industries. Dry products typically exhibit high quality, low levels of degradation, and excellent stability properties.

Carbohydrate polymers are naturally occurring molecules that exhibit similar behavior to synthetic polymers, i.e., with properties such as glass transition temperature (Tg), melting temperature (Tm), and molecular weight distribution (MWD). These polymers are mainly based on polysaccharides composed of glucose, sucrose, dextrose, arabinose and galactose; examples of these include starch, chitosan, maltodextrins (MX), inulin (IN), and gum Arabic (GA). Polysaccharides have been widely used individually as carrier agents for the conservation and microencapsulation of active ingredients in food and pharmaceutical products. For example, chitosan has been used in the preservation of meat products [9] and as a protective barrier in edible films [10]. MXs with different MWD have been compared as carrier agents in the yield, and content of natural antioxidants such as quercetin and resveratrol [11,12]. IN has also been employed as a carrying agent in the spray-drying preparation of a functional food based on orange juice [13]. MX and IN were compared in the conservation of antioxidants from blueberry juice, showing that low molecular MXs presented a higher retention value than IN [14]. Recently, the performance of IN and lactose as carrying agents in the co-microencapsulation of *Bacillus clausii* (Bc) and resveratrol as active ingredients of a functional food was reported [15]. The results showed higher antioxidant activity for IN, and similar cell viability for both wall materials.

Regarding the use of blends of carrying agents, most of the works are based on polysaccharide-protein complexes coacervates [16]. Bordón et al. [17] reported a higher encapsulation efficiency of chia oil when using soy protein isolate and GA blends at a ratio of 2:1. Guo et al. [18] employed a surfactant (Rhamnolipid) to fabricate complexes of pea protein isolate (PPI) and high methoxyl pectin (HMP) and studied its effect on the releasing of curcumin and resveratrol. They concluded that the use of the surfactant improved the encapsulation efficiency, enhanced the solubility in water and delayed the delivery of the antioxidants. Kumar and Saini [19] prepared edible bi-layer coatings for prolonging the shelf life of tomatoes, employing whey protein isolate (WPI), xanthan gum and clove oil. They found that the decrease in the total phenolic content was considerably lower on the coated fruits than on those uncoated. Sharifi et al. [20] reported the improvement in cell viability of *Lactobacillus plantarum* when adding phytosterols to a blend of WPI and GA.

Conversely, other works have reported the synergistic effect of using two or more polysaccharide components. Maisuthisakul and Gordon [21] studied the combination of GA, MX, and alginate (AL) on the encapsulation efficiency of phenolic compounds and the storage properties of mango seed kernel emulsions. The optimal mixture for these properties was composed of 5.95% of GA, 23.9% of MX, and 0.11% of AL. de Barros Fernandes, Vilela Borges, and Alvarenga Botrel [22] tested the replacement of GA by modified starch (MS), MX, and IN on the microencapsulation properties of rosemary essential oil. The highest oil retention (60%) was obtained when employing a 1:1 mixture of MS and MX. Silva and Hubinger [23] employed mixtures of MS or GA with MX at a ratio of 75:25 for the microencapsulation of green coffee oil. The highest encapsulation efficiency of 87.6% was achieved with the MS-MX blend. Damodharan et al. [24] employed AL and two gums to test the viability of microencapsulated bacteria. The composition of 1% AL, 0.5% Fenugreek gel and 0.5% Locust bean gum was selected based on the viscosity of the mixture, because this property directly affects the spray drying process. For this mixture, the survival rate of the bacteria was greater than 85% and 97% in simulated gastrointestinal and colonic fluids, respectively. Poletto et al. [25] compared the use of IN, rice bran, and starch (Hi-maize) on the encapsulation of *L*. *acidophilus* as a probiotic. The highest microencapsulation efficiency (96%) was presented by the blend containing 2% of AL and 10% of IN. Colín-Cruz et al. [26] employed GA, MX and whey protein concentrate (WPC) to prepare 50–50% blends. They found that the GA-MX blend increased the encapsulation efficiency of the phenolic compounds and anthocyanins to 98.4% and 99%, respectively, while the single WPC showed a higher encapsulation efficiency for the probiotic bacteria.

Determining the effects of the processing conditions on the physicochemical and functional performance of powder products containing microencapsulated active ingredients is essential for novel foods and pharmaceutical products development. Active ingredients, such as quercetin and *Bacillus claussi* microorganisms are sensitive to degradation by environmental conditions including temperature, humidity, and exposure to light and oxygen. Therefore, the co-microencapsulation of these ingredients is essential to maintain the antioxidant and probiotic properties during the storage of the powder product.

Derived from the above, the present work aims to obtain a functional food powder, with probiotic and antioxidant properties, based on mixtures of carbohydrate polymers such as MX and IN. For this purpose, a reduced design of experiments was developed to study the effect of the spray drying inlet temperature, and the concentration of the polysaccharides on the morphology, microstructure and Tg of the obtained powders, as well as their antioxidant activity and cell viability. In this sense, herein is demonstrated the beneficial use of blends of carbohydrate polymers, and how these polysaccharides affect the studied properties. The work contributes to the technological application of carbohydrate polymers as functional food matrices, the co-microencapsulation and conservation of active ingredients, and the preparation of a functional food powder.

## 2. Materials and Methods

### 2.1. Materials

The following reagents were purchased from the specified vendor. Commercial maltodextrin (MX) extracted from cornstarch, and inulin (IN) (Ingredion, Guadalajara, Mexico). The dextrose equivalent (DE) of MX was 10, corresponding to a molecular weight of 1625 g/mole and a degree of polymerization (DP) of 2–16 units of glucose. *Bacillus* bacteria (Bc) strain (*Bacillus clausii*) in Sunuberase solution (Sanofi-Aventis, Coyoacán, CDMX, Mexico). Quercetin 3-d-Galactoside (99%, Química Farmacéutica Esteroidal, Tláhuac, CDMX, México). Analytical grade 2,2-diphenyl-1-picrylhydrazyl (DPPH), (±)-6-hydroxy-2,5,7,8-tetramethylchromane-2-carboxylic acid (Trolox), gallic acid, sodium carbonate (Na_2_CO_3_), and Folin–Ciocalteu reagent (Sigma–Aldrich Chemical Co., Toluca, Mexico).

### 2.2. Preparation of Spray-Dried Powders

Spray drying process was employed in the microencapsulation of Bc and quercetin in the form of powders. Typically, the preparation of the feeding solutions consisted of mixing 20 g of the corresponding carrying agent (inulin, maltodextrin or the blend), 1 g of quercetin, 5 mL of the commercial solution with bacteria (equivalent to a concentration of 2 × 10^12^ CFU), and distilled water for obtaining a total volume of 100 mL of solution. Microencapsulation was carried out in a Mini Spray Dryer B290 (BÜCHI, Labortechnik AG, Flawil, Switzerland) at the following operation conditions: feed temperature of 40 °C, feeding flow of 7 cm^3^/min, hot airflow of 28 m^3^/h, aspiration of 70%, and pressure of 1.5 bar. The inlet temperatures were varied in the range of 150–220 °C. This range of temperatures was selected to avoid the collapse of the microstructure and other unwanted characteristics such as stickiness and agglomeration of the powders.

A reduced design of experiments was carried out to test the effect of the inlet temperature and the concentration of the carrying agents in the blends. Through the coding of parameters ranging from –1 for the lowest value, and 1 for the highest value, robustness and reliability are obtained in the acquired data. The experiment design contains points outside the surface (±1.4) as control points to carry out interpolations of the variables, verify the reliability and establish inflection points. Table 1 shows the run number and the corresponding experimental conditions. Once the powders were obtained, these were individually placed in airtight bags and stored in darkness at 4 °C. Each sample was labeled as INMX_x, where IN and MX stand for the carbohydrate polymer employed as the carrying agent in the blend, and x stands for the run number.

### 2.3. Scanning Electron Microscopy (SEM)

Morphological characterization was conducted using a scanning electron microscope (SEM) (JSM-7401F, JEOL, Tokyo, Japan) operated at an accelerating voltage of 2 kV. Powder samples were first dispersed on a double-side copper conductive tape, then covered with a thin layer of gold utilizing a sputtering to reduce charging effects (Denton Desk II sputter coater, Denton, TX, USA).

### 2.4. X-ray Diffraction (XRD)

Microstructural characterization was determined by x-ray diffraction (XRD) analysis in a D8 Advance ECO diffractometer (Bruker, Karlsruhe, Germany) equipped with Cu-K radiation (l = 1.5406 Å) operated at 45 kV, 40 mA and a detector in a Bragg–Brentano geometry. Scans were performed in the 2θ range of 5–50°, with a step size of 0.016° and 20 s per step.

### 2.5. Thermal Analysis

A modulated differential scanning calorimeter (MDSC) Q200 (TA Instruments, New Castle, DE, USA) equipped with an RCS90 cooling system was employed for determining the Tg. The instrument was calibrated with indium for melting temperature and enthalpy, while sapphire was used as the standard for heat capacity (Cp). Samples about 10 mg were encapsulated in Tzero aluminum pans. Thermograms were acquired at a temperature range of −50 to 250 °C, with a modulation period of 40 s and amplitude of 1.5 °C.

### 2.6. Determination of Water Activity

For each of the microencapsulated powders, water activity (aw) was determined into an Aqualab Series 3 Water Activity Meter (Meter Group, Inc. Pullman, WA, USA). According to the AOAC, the method requires drying the sample in an oven at 110 °C for 2 h, and calculating the ratio of the final and initial masses.

### 2.7. Radical Scavenging Activity of the Functional Food

The antioxidant capacity of microencapsulated quercetin was determined according to the methodology reported in [15,27]. The microencapsulated powders were dissolved in ethanol at concentrations of 5, 10 and 30 μg/mL. Then, 1.7 mL of each solution was mixed with 1.7 mL of DPPH* ethanol solution (0.1 mmol/L). The initial powder concentration in solution were 2.5, 5 and 15 µg/mL, while the DPPH* solution concentration decreased to 0.05 mmol/L. The mixed solution (3.4 mL) was poured into a 10 mm thick quartz cell. The antioxidant capacity was evaluated as the decrease in the initial concentration of DPPH* scavenged by quercetin after 30 min of preparation. The variation in the absorbance intensity of DPPH* was measured at a wavelength of 517 nm in a UV-Vis spectrophotometer Evolution 220 (Thermo Scientific, Waltham, MA, US). The scavenging activity (%DPPH*) was calculated according to Equation (1):

(1) $$\mathrm{Scavenging}\;\mathrm{Activity}\;\left(\%\;\mathrm{DPPH}\right)=\frac{A_{0}-A_{30}}{A_{0}}\times 100$$

where A_0_ is the initial absorbance of DPPH*, and A_30_ is the absorbance of the DPPH* after 30 min of adding the microencapsulated antioxidant.

### 2.8. Viability of Bacillus Clausii in the Microencapsulated

The number of available Bc bacteria cells was evaluated by means of the plate extension technique, with Trypticase-Soy Agar (TSA) (Becton Dickinson, Franklin Lakes, NJ, US), using serial dilutions of the encapsulated samples from 1 × 10^−1^ to 1 × 10^−7^. Growing conditions were aerobic, with an incubation period of 48 h at 37 °C in an incubator (Novatech, Jalisco, GDL, Mexico). To determine the number of colony-forming units per gram (CFU/g), the concentrations exhibiting between 300 and 30 CFU (1 × 10^−4^ and 1 × 10^−5^) were selected. Equation (2) was employed for the quantification of culturability. All experiments were performed by triplicate, and the reported values represent the average of the calculated values.

(2) $$\frac{\mathrm{CFU}}{g}=\left[\frac{N{}^{\circ}\;\mathrm{plate}\;\mathrm{colonies}\;\times \;\mathrm{dilution}\;\mathrm{factor}}{\mathrm{mL}\;\mathrm{sample}\;\mathrm{seeded}}\right]$$

### 2.9. Powder Yield

Yield percentage (%) was calculated according to Equation (3), by the ratio of the masses of the collected powder (W_P_), and the liquid (W_L_) fed into the spray dryer.

(3) $$Y\left(\%\right)=\frac{W_{P}}{W_{L}}\times 100$$

### 2.10. Encapsulation Efficiency (EE)

The total phenolic content (TPC) and the surface phenolic content (SPC) were determined by the modified Folin–Ciocalteu method [28]. For TPC, 50 mg of microcapsules were weighed and dissolved in a 1 mL solution of ethanol-acetic acid-water (50:8:42 *v*/*v*). For SPC 50 mg of microcapsules were dispersed with a 1 mL solution of ethanol-methanol (1:1 *v*/*v*). Both mixtures were agitated using magnetic stirring for 1 min and centrifuged at 11,000 rpm by 15 min at 10 °C. Absorption at 750 nm was measured using a UV/VIS spectrometer. Calibration curves were prepared with different quercetin concentrations in ethanol-acetic acid-water solution, or ethanol-methanol solution. TPC and SPC were expressed as quercetin equivalents (QE) in milligrams per gram of microencapsulates.

The encapsulation efficiency (EE) was expressed as the ratio of encapsulated phenolic content (EPC) to total phenolic content (TPC). EPC was determined as the difference between TPC and SPC. The encapsulation efficiency of microcapsules was calculated according to Equation (4).

(4) $$\mathrm{EE}\left(\%\right)=\left(\frac{\mathrm{TPC}-\mathrm{SPC}}{\mathrm{TPC}}\right)\times 100$$

### 2.11. Statistical Analysis

All experiments were performed by triplicate, reporting mean values and standard deviations. A one-way analysis of variance (ANOVA) was performed to establish a significance level of 0.05, and the Tukey’s honestly significant difference (HSD) post hoc test was used to determine the difference between the means. The statistical analyses were conducted using the IBM SPSS statistics version 21.0 software (SPSS Inc., Chicago, IL, USA).

## 3. Results and Discussion

### 3.1. Morphological Characterization

The overall appearance of the spray-dried powders was a yellowish color composed of fine non-aggregated particles, suggesting that the processing conditions were adequate to avoid the collapse of the microstructure and the appearance of undesired drying characteristics such as agglomeration, stickiness, and crystallization [29].

**Figure 1:**
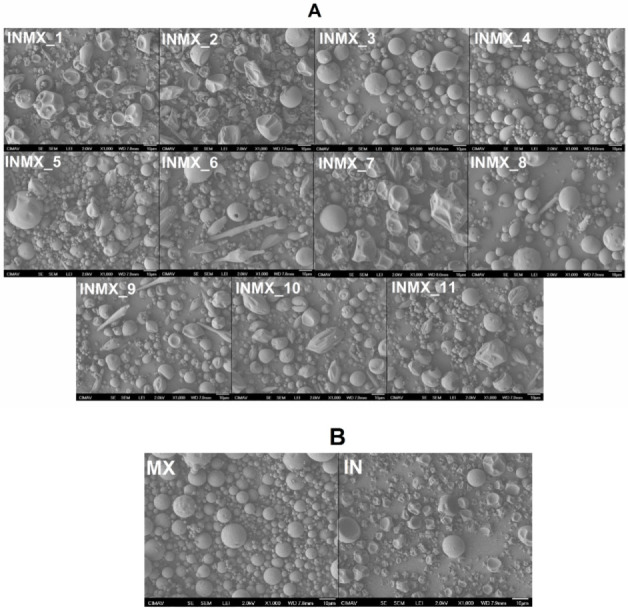
Acquired SEM micrographs at 1000× of the spray-dried powders: (**A**) carbohydrate polymers blends, and (**B**) blank samples.

Figure 1 shows SEM micrographs acquired at 1000× of the spray-dried powders. By comparing the prepared blends (samples INMX_1–11 in Figure 1A) against the single MX and IN blank samples (Figure 1B), is possible to observe the following features. (i) The morphology of the particles was in general pseudo-spherical, but at some processing conditions, the shape was spheroidal and irregular as deflate balls. In some cases, a third morphology composed of elongated fiber-like or rod-like particles was observed. (ii) The particle size was in the order of the micrometers with at least two size distributions: large particles of a few tens of microns, and small particles below 5 microns. (iii) The surface of the particles was smooth at some processing conditions and wrinkled at others. (iv) Bacteria were not observed on the particles or in between, suggesting that they were completely microencapsulated or fully eliminated by thermal degradation during the spray drying process.

By relating the processing conditions with the above-mentioned observations, it seems that the morphology of IN particles was deformed with the increase in the drying temperature (INMX_8, 4, and 11). On the other hand, the MX particles were relatively stable at the intermediate and higher drying temperatures (INMX_1, 6, and 10), since at these temperatures (180 and 210 °C) the morphology of the particles was relatively more regular than at 150 °C. The observed surface appearance of deflated balls or wrinkle surfaces is caused by the rapid removal of water during drying. Additionally, the lack of surface cracks on the particles is important to reduce gas permeability and, to promote the conservation of the active ingredients within the walls of the particles [22], and it indicates the complete coverage of the active materials by the polysaccharides [23]. Araujo-Díaz et al. [14] showed the variation in the morphology of particles with the adsorption of moisture of spray-dried MX and IN powders. They found that the morphology of the particles was initially spherical, while after the powders were exposed to different water activities, the particles started to coalesce and grow with irregular morphologies.

Although in some studies, the decrease in the size of the particle has been related to the addition of a second carrier agent [26], in the present work the size of the particles increased with the addition of the two polysaccharides. For example, at the same drying temperature (180 °C) the average sizes and standard deviations of the IN, MX and blend samples (INMX_4, 6 and 2) were 3.2 ± 2.57 μm, 4.1 ± 2.95 μm, and 8.1 ± 5.05 μm, respectively.

Related to whether or not bacteria are present in the functional powdered food, by comparing the morphology of the particles in the micrographs, in the blank samples (Figure 1B) is observed the presence of spherical and pseudo-spherical particles, and the absence of particles with elongated rod-like morphologies. This suggests that the *Bacilus claussi* microorganisms were microencapsulated with the corresponding polysaccharide in the 11 runs (Figure 1A), and formed this type of elongated particles. Conversely, several studies where the organisms were not observed either, have reported that the morphology of the particles was not modified with the addition of bacteria [15,20]. However, in these works, these powders showed significant bacterial activity.

The morphological characterization suggested that the obtained powders herein were fully dried and that the active ingredients, i.e., antioxidant and microorganisms were successfully microencapsulated within the pseudo-spherical particles and the rod-like particles, respectively.

### 3.2. Microstructural Characterization

**Figure 2:**
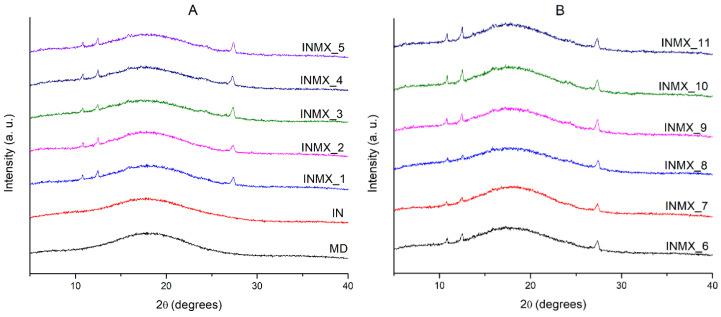
X-rays diffractograms of the spray-dried powders containing the active ingredients: (**A**) IN and MX blanks, and blends INMX_1–5. (**B**) Blends INMX_6–11.

The microstructure of the powdered functional food was studied by XRD. Figure 2 shows the X-rays diffractograms of the spray-dried powders containing the active ingredients and the blank samples. The diffractograms of the blank samples (MX and IN) showed a single low intensity and broad peak about an angle 2θ of 18°, and the absence of other diffraction peaks. This indicated that in both polysaccharides the microstructure was completely amorphous after the spray drying process. MX and IN are based on carbohydrate polymers composed of chains of glucose and fructose, respectively. In consequence, their microstructure and properties are different. For example, when these polysaccharides are individually subjected to the adsorption of moisture, the MX powder does not crystallize, but just changes its microstructure from the amorphous into the rubbery state at low and intermediate water activities, and into a liquid state at high water activities [30]. Meanwhile IN modifies its microstructure from the amorphous state into a crystallized matrix, showing a corresponding increase in the intensity of the diffraction peaks at 2θ of 12, 16, 18.5, and 23° [29].

On the other hand, the samples containing the active ingredients (INMX_1–11) besides the amorphous broad peak at 18°, presented three notable diffraction peaks at 2θ angles of 10.8, 12.4 and 27.3°. This observation is interesting because it could suggest the crystallization of the carrying agents during the spray drying. However, according to the design of experiments (Table 1) several of the samples were prepared as a mixture of two polysaccharides, while others contained only one carrying agent. Therefore, from these observations it can be deduced that some of the active ingredients are crystallizing during the drying. Recently, Klitou et al. [31] reported the simulated and experimental XRD diffractograms of quercetin crystallized in dimethyl sulfoxide (DMSO), identifying characteristic diffraction peaks at 2θ angles of 10.5, 12.9, 16.3, 17.1, 18.3, 19.5, 20.4, 23.2, 27.2, and 27.8°. Morphological observations from SEM could suggested that the peaks in diffractograms are caused either by the non-spherical shaped particles such as elongated fiber-like particles or by the crystallization of quercetin.

### 3.3. Thermal Analysis

**Figure 3:**
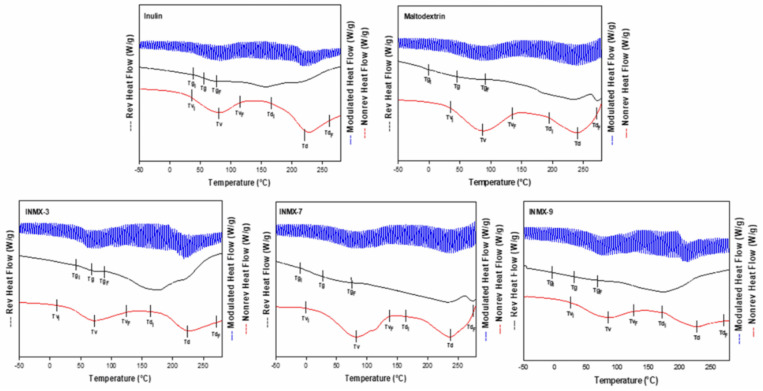
MDSC thermograms of selected spray-dried powders containing the active ingredients (INMX_3, 7, and 9) and the blank samples (MX and IN).

The determination of Tg is very important because this value indicates the transition from the vitreous state into the rubber state. Commonly, the powders obtained by spray drying are in the vitreous state, in which the mobility of polysaccharides chains is restricted, limiting the diffusion phenomena of water molecules. Macroscopically, this is observed as a well-dispersed and fine powder i.e., without forming aggregates or caking. Additionally, the Tg values have been related to the stability during storage of food products [32], in such a way that carrying agents with higher Tg produce powders with greater stability during processing and storage. When the Tg value is close or below the ambient temperature, the system may experience a thermodynamic imbalance, modifying its microstructure from the amorphous into an intermediate rubber or crystalline state. Thus, the selection of carrier agents is important since the final Tg of the mixture will depend on factors such as molecular weight, water content, and chemical compatibility [22]. Figure 3 shows the MDSC thermograms of selected spray-dried powders containing the active ingredients (INMX_3, 7 and 9) and the blank samples (MX and IN). In each graph are plotted three curves: on top (blue color) the total heat flow curve, in the middle (black color) the reversible heat flow curve, and on the bottom (red color) the non-reversible heat flow curve. Additionally, on each curve are indicated the beginning (T_i_), the peak value, and the ending (T_f_) of the identified thermal event.

The summary of the Tg values is presented in Table 2. The Tg values were determined in the range of 15.9–110.2 °C, while a_w_ was 2.02–5.50. In all cases, the Tg decreased monotonically with increasing a_w_. These results are very similar to those reported by Vera Lavelli et al. [33] who obtained encapsulated phenols from grape skin with maltodextrin (GPS) with different aw values. They observed the formation of lumps at 19% of moisture and a_w_ of 0.75, and a viscous liquid at 12% of moisture and a_w_ of 0.56. Bordón et al. [17] reported a similar behavior for blends of SPI-GA prepared at 1:1 and 2:1 ratios. From the prepared powders, the blend dried at 234 °C (INMX_3) showed the highest Tg value (60.7 °C), while the rest of the blends presented values below 30 °C. Thus, if the functional food is to be stored at room temperature (25 °C), it is probable that the blend dried at 234 °C may present the greatest stability than the rest of the tested blends. The maximum stability of food occurs when the storage temperature is lower than the Tg. Colín-Cruz showed that the addition of other carbohydrate polymers increased the Tg from 46.1 °C to 51.6 °C for WPC and GA-WPC, respectively [26].

From the previous physical characterization, it is evident that the spray drying process promoted the obtaining of a powder functional food with particle size and morphology for avoiding particle aggregation, an amorphous microstructure to retain and preserve the active ingredients, and different Tg values to tailor different stabilities during storage.

### 3.4. Antioxidant Activity

**Figure 4:**
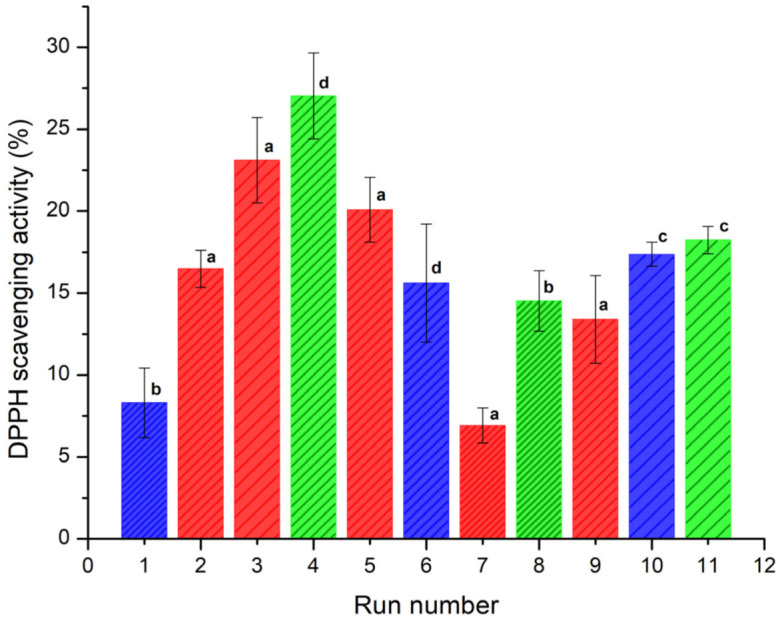
DDPH scavenging capacity (%) of the spray-dried powders containing the active ingredients (INMX_1–11) at 30 min of exposition. Bar color indicates the polysaccharide group (blue for MX, green for IN, and red for blends). Pattern thickness indicates the drying temperature (dense for low temperature, medium for intermediate temperature, and sparse for high temperature). Lowercase letters indicate the Tukey’s test results at significance level of 0.05. a: significance difference, effect of drying temperature; b: significance difference, effect of composition at 150 °C; c: no significance difference, effect of composition at 210 °C; d: significance difference, effect of composition at 180 °C and 28.4% solids.

The antioxidant activity (AA) was determined in terms of radical-scavenging using the stable DPPH radical. Reduction of DPPH• by an antioxidant (DPPH• + A → DPPH-H + A•) or by a radical species (DPPH• + R• → DPPH-R) results in a loss of absorbance at 515 nm [34]. The AA was determined at concentrations of 2.5, 5 and 15 μg/mL. The results showed a noticeable increase in the AA at 15 μg/mL for all the samples. Figure 4 shows the DDPH scavenging activity (%) of the spray-dried powders containing the active ingredients (INMX_1–11). The observed antioxidant activity was related to the content of quercetin released after 30 min of exposition. Evidently, the ability to scavenge free radicals increased with the amount of quercetin released. The AA values are presented in Table 2. Run 7 (INMX_7) showed the lowest AA value of 6.91%, while run 4 (INMX_4) had the highest value of 27.03%. These experiments corresponding to the 50-50 blend of IN-MX dried at 126 °C, and to the IN dried at 180 °C with the highest concentration of solids (28%), respectively. By comparing the single carrying agents, IN showed a higher AA than MX at all the drying temperatures. This suggested a tight binding between MX and quercetin, that retains quercetin bioactivity from interacting with free DPPH radicals.

For the microencapsulates prepared with carbohydrate blends (INMX_2, 3, 5, 7, and 9) the AA values varied in the range of 6.91–23.11%, increasing with the drying temperature, and with the content of IN.

IN microencapsulates at the highest concentration (28%) produced the highest antioxidant activity, while at the concentration of 20% (INMX_8 and 11) the AA values were 14.52 and 18.24%. This behavior resulted from the number of dissolved solids in the solution, where quercetin was mixed with the polysaccharide. However, when comparing the samples with the same concentration of solids, i.e., 20%, it is observed that blends INMX_3 and 5 presented higher AA values than those of single IN. Additionally, the IN presents a cost in the market approximately four times higher than the MX. Therefore, reducing the IN content in the food product while maintaining the AA at a high level is beneficial. These observations suggested a synergistic effect on the microencapsulation and conservation of antioxidants when mixing two polysaccharides such as IN and MX.

### 3.5. Culturability of B. clausii after the Spray-Drying Process

**Figure 5:**
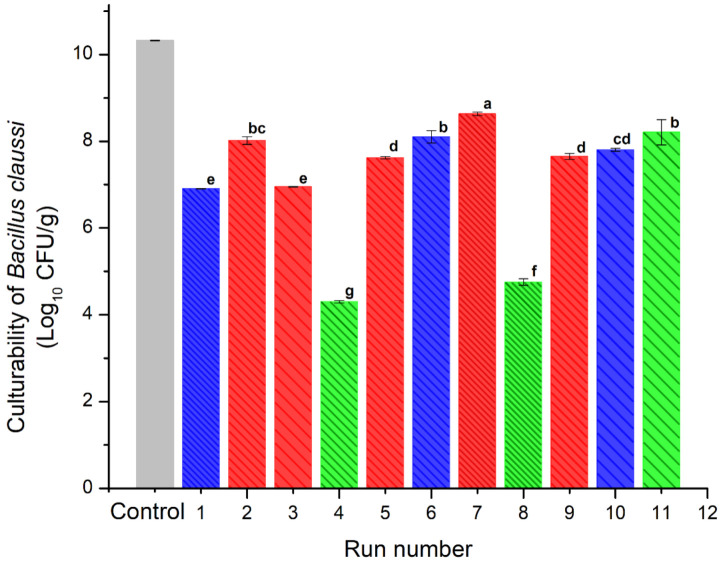
Culturability of *B. clausii* of the spray-dried powders containing the active ingredients (INMX_1–11). Bar color indicates the polysaccharide group (blue for MX, green for IN, and red for blends). Pattern thickness indicates the drying temperature (dense for low temperature, medium for intermediate temperature, and sparse for high temperature). Lowercase letters (a–g) indicate the Tukey’s test results at significance level of 0.05.

Figure 5 shows the results obtained for the survival rate of *B. clausii* microencapsulated after the spray drying process. Each treatment was inoculated with a population of 10.32 log CFU/g of lactobacillus. All treatments presented at least one log reduction cycle due to the spray drying process. Equal to our results, Paim et al. [35] reported that spray drying caused a reduction near of 1 log cycle in *Bifidobacterium* spp. Lactis encapsulated at temperatures similar to those employed in this work.

The blends presented similar viability values in the range of 7–8.6 log CFU/g. The highest viability value was presented by the sample dried at the lowest temperature (INMX_7). Except for sample INMX_8 with a viability value of 8.3 log CFU/g, the other samples containing only IN (INMX_4 and 11) presented the lowest cell viability values of 4.2 and 4.6 log CFU/g, respectively. On the other hand, the samples containing only MX presented values between 6.9–8 log CFU/g.

These observations indicated that at low drying temperatures, i.e., 150 °C, IN is more efficient as a microencapsulating agent for the microorganism. Meanwhile, MX performs well as a protective agent for microorganisms in a wide range of temperatures, for example 150–210 °C. In the case of the blends, the addition of MX was beneficial, since it promoted the microencapsulation and conservation of the microorganism in the presence of IN, maintaining relatively high viability throughout the entire range of temperatures tested.

Pandey and Mishra [36] encapsulated *L. plantarum* by spray drying in a matrix composed of inulin, dextran, and maltodextrin. They obtained an optimum composition of 0.4%, 4.6%, and 8.4% of inulin, dextran, and maltodextrin, respectively. Although inulin provides favorable conditions for the growth and viability of probiotics, the *Bc* microorganisms did not efficiently metabolize the long carbohydrate chains composing the microstructure of IN, and consequently did not increase their viability.

Based on the cell viability results, all of the spray-dried samples prepared in this work (with exception of runs INMX_4 and 11) may be considered as powders with probiotic properties. The powders presented viabilities higher than 6 log CFU/g, which is recommended by FAO/WHO (2003) as the minimum value required to produce therapeutic benefits. Although the use of inulin as single microencapsulating material is not adequate for the survival of the lactobacillus, its addition in the blend may be beneficial as an aid in the spray drying, increasing the overall Tg of the powder and reducing processing problems such as stickiness [37,38].

### 3.6. Effect of the Processing Conditions on the Yield

**Figure 6:**
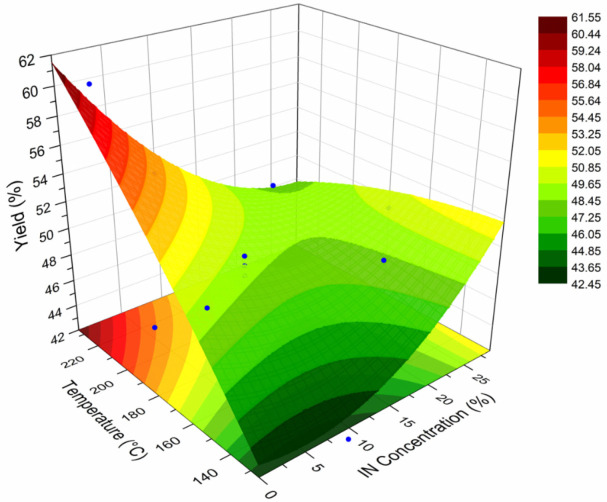
SRP for the yield of the spray-dried powders containing the active ingredients (INMX_1–11).

A surface response plot (SRP) is a graphical representation of the effect of two experimental variables on a given response variable. These plots have been employed to describe the behavior of polymeric systems under distinct processing conditions [11,39]. In this sense, a SRP was constructed to relate the effect of the spray drying temperature and concentration of IN on the yield (%) of powder obtained. The calculated values of the yield are included in Table 2, while Figure 6 presents the SRP for the yield of the spray-dried powders containing the active ingredients (INMX_1–11). In the *X*-axis is plotted the drying temperature, in the *Y*-axis the IN concentration, and in the *Z*-axis the yield. In the IN concentration axis, the concentration values of cero corresponds to the runs containing only MX (INMX_1, 6 and 10). The IN concentration value of 10% correspond to the blends (INMX_2, 3, 5, 7, and 9), while the concentration values of 20% and 28% belong to the runs containing only IN (INMX_4, 8 and 11). At the lowest concentration value, the yield increased almost linearly with the drying temperature. The highest yield value of 61% was obtained for the run INMX_10, processed at 210 °C and with 20% of MX. The blends showed an increase in the yield with the temperature, reaching a maximum value at the intermediate drying temperature (180 °C), observed as a flat region in the center of the surface. At the high IN concentrations, the yield remained almost unchanged with the temperature. Another noticeable feature observed in the SRP is the color of the fringes, which correspond to different yield values. These fringes are helpful to identify similar yield values obtained at distinct processing conditions. For example, at the temperature of 160 °C and single MX, or a temperature of 150 °C and IN concentration of 25%, either condition may be selected to obtain a yield of 48–49% (central green fringe).

The calculated predicting model derived from the non-linear surface fit is presented in Equation (5):

Y(%) = 13.7401 + 0.26271T + 0.75803C − 2.5014 × 10^−4^T^2^ + 0.01636C^2^ − 0.0073T × C (5)

where Y is the predicted yield (%), T the drying temperature (°C), and C is the IN concentration (%). With this model is possible to predict the value of the yield at different processing conditions of T and C. However, the model is only valid within the values of the processing conditions tested herein.

The encapsulation efficiency (EE) of the encapsulated quercetin in INMX_1–11 blends is shown in Table 2. EE varied in the range of 7–96%, corresponding to a quercetin content of 0.53–12.47 mg of quercetin per g of powder. Single MX presented largest EE values at intermediate and highest drying temperatures, than single IN at the highest drying temperature. The INMX blends presented higher EE values than the single MX and IN powders. In addition, the INMX_3 and 7 showed the highest EE values, corresponding to the highest and lowest drying temperatures, respectively.

Cilek et al. [28] extracted and encapsulated the phenolic compounds from sour cherry pomace by freeze-dried. They employed MX and GA at ratios of 1:10 and 1:20, and 10% of solids concentration. The powders with only MX presented an EE of 69.38%, while for the blends, the EE increased up to 92.26% when increasing the GA content to a ratio of 6:4. The same behavior was observed in the current investigation, where higher EE was obtained when employing 1:1 blends of IN and MX.

Etzbach et al. [40] microencapsulated golden berry juice by spray drying using six different carrier agents and blends at 140 °C of inlet temperature and 20% of solids concentration. They determined carotenoid retention in the range of 16.4–77.2%, and attributed this behavior to different particle morphologies and drying rates caused by the viscosity of the feed suspension. The extent of the spray drying process depends on the viscosity of the feed suspension. During the spray drying, the first drying phase is formed at lower temperatures, while the second drying phase or crust is formed at higher temperatures. Highly viscous feed suspensions such as those containing AL and GA provoke circulation currents within the droplet, promoting a rapid crust formation of the first drying phase. Low viscous feed suspensions such as those containing MX reduce the exposure to high drying temperatures since the first drying phase of crust formation is extended.

## 4. Conclusions

A powdered functional food based on blends of carbohydrate polymers was prepared by spray drying. The resulting powders were composed of inulin (IN), maltodextrin (MX), and active ingredients with antioxidant and probiotic properties such as quercetin and *Bacillus clausii*, respectively. The physical characterization showed a non-aggregated powder with pseudo-spherical shape particles of micrometric sizes. The observation of rod-like particles in the blends and not in the single polysaccharides, suggested that the microorganisms were microencapsulated in these type of particles. An amorphous microstructure indicated the microencapsulation of the active ingredients within the particles, while the diffraction peaks suggested the crystallization of quercetin. The determined Tg values were above the room temperature, which may promote the stability of the powder during storage. The antioxidant activity and viability showed synergistic effects with the IN-MX blends. The antioxidant activity increased with the addition of IN, while the viability increased with the addition of MX. A response surface plot (RSP) was constructed for the yield of the functional food powder. The yield of the blends was slightly more affected by the drying temperature than by the IN concentration. This RSP was useful to identify regions of similar yield values at different processing conditions. The INMX blends presented higher encapsulation efficiency (EE) values than the single MX and IN powders.

This work showed the beneficial use of blends of carbohydrate polymers for the microencapsulation and preservation of active ingredients for food and pharmaceutical applications.

## Figures and Tables

**Table 1:** Reduced experimental design.

Run	Order	Temperature	Carrying Agent	Temperature (°C)	MD (%)	IN (%)
1	1	−1.000	−1.000	150	20	0
2	9	0.000	0.000	180	10	10
3	6	1.414	0.000	234	10	10
4	8	0.000	1.414	180	0	28.8
5	10	0.000	0.000	180	10	10
6	7	0.000	−1.414	180	28.8	0
7	5	−1.414	0.000	126	10	10
8	3	−1.000	1.000	150	0	20
9	11	0.000	0.000	180	10	10
10	2	1.000	−1.000	210	20	0
11	4	1.000	1.000	210	0	20

**Table 2:** Summary of the results of the spray-dried powders containing the active ingredients.

Run	Tg (°C)	a_w_	AA (%)	*Bc* (Log_10_ CFU/g)	Yield (%)	EE (%)
1	35.2	2.42	8.3 ± 2.13	6.90845 ± 0.007	51.09	7.48 ± 0.16
2	28.1.8	2.77	16.48 ± 1.12	8.01671 ± 0.088	48.95	29.50 ± 0.42
3	60.7	2.41	23.11 ± 2.6	6.95422 ± 0.006	52.09	96.49 ± 0.64
4	110.2	4.48	27.03 ± 2.63	4.30049 ± 0.03	49.1	80.71 ± 0.12
5	24.3	4.48	20.08 ± 1.98	7.62276 ± 0.029	48.19	17.84 ± 0.77
6	39.7	2.45	15.61 ± 3.6	8.10206 ± 0.144	47.09	46.34 ± 0.14
7	15.9	5.22	6.91 ± 1.07	8.63241 ± 0.042	41.14	93.26 ± 0.68
8	51.1	4.81	14.52 ± 1.85	4.75257 ± 0.075	49.28	83.02 ± 0.42
9	25.9	4.01	13.39 ± 2.68	7.65051 ± 0.068	49.66	29.21 ± 0.57
10	40.1	2.02	17.36 ± 0.74	7.80211 ± 0.033	61.28	43.25 ± 0.47
11	38.4	5.50	18.24 ± 0.83	8.20749 ± 0.293	50.71	26.62 ± 0.34

(Tg) glass transition temperature, (a_w_) water activity, (AA) antioxidant activity, (*Bc*) viability of *Bc* bacteria, and (EE) encapsulation efficiency.

## Data Availability

Data are contained within the article.
